# A Randomized Controlled Trial: Evaluating the Sleep, Cancer and Rest (SleepCaRe) Trial to Improve Health‐Related Quality of Life in Women Undergoing Chemotherapy for Breast Cancer

**DOI:** 10.1002/pon.70418

**Published:** 2026-03-22

**Authors:** Rebecca Wallace, Marliese Alexander, Daphne Day, Justine Diggens, Maria Ftanou, Veronica Aedo‐Lopez, Bei Bei, Robert Blum, Frances Boyle, Trang Thuy Do, Prudence A. Francis, Sheila N. Garland, Jordan Maccora, Sharad Sharma, Lesley Stafford, Michelle White, Duncan Mortimer, Joshua F. Wiley

**Affiliations:** ^1^ School of Psychological Sciences Monash University Melbourne Australia; ^2^ Pharmacy Department Peter MacCallum Cancer Centre Melbourne Australia; ^3^ Sir Peter MacCallum Department of Oncology The University of Melbourne Parkville Australia; ^4^ Department of Oncology Monash Health Melbourne Australia; ^5^ Department of Medicine, Nursing and Health Sciences Monash University Melbourne Australia; ^6^ People and Culture Peter MacCallum Cancer Centre Melbourne Australia; ^7^ Department of Psychology Psychosocial Oncology Program Peter MacCallum Cancer Centre Melbourne Australia; ^8^ Department of Oncology Bendigo Cancer Centre Bendigo Health Bendigo Australia; ^9^ Patricia Ritchie Centre for Cancer Care and Research Mater Hospital North Sydney Australia and University of Sydney Sydney Australia; ^10^ Department of Medical Oncology Peter MacCallum Cancer Centre Melbourne Australia; ^11^ Department of Psychology Memorial University St. John's Newfoundland and Labrador Canada; ^12^ Department of Medical Oncology Ballarat Regional Integrated Cancer Centre Grampians Health Ballarat Australia; ^13^ Department of Surgery University of Melbourne Melbourne Australia; ^14^ Genomic Medicine and Parkville Familial Cancer Centre The Royal Melbourne Hospital Parkville Australia; ^15^ Centre for Health Economics Monash University Melbourne Australia

## Abstract

**Purpose:**

Advances in cancer treatment have led to improved survival rates, but challenges related to health‐related quality of life (HRQoL) persist, often exacerbated by sleep disturbances. We present a pre‐registered, secondary analysis of HRQoL from a trial of sleep interventions among women with early or advanced breast cancer receiving chemotherapy.

**Patients and Methods:**

This 6‐week, multisite, remotely‐delivered, randomized controlled trial compared Cognitive Behavioral Therapy for Insomnia (CBT‐I), Bright Light Therapy (BLT), their combination (CBT‐I + BLT), and an active control (Sleep Hygiene Education, SHE) on HRQoL as measured by the PROMIS‐Preference score (anchors: 0 “Dead” to 1 “Full health”) at baseline, mid‐point (3‐weeks), post‐intervention (6‐weeks), and follow‐ups (3&6‐months). Interventions involved emails and tailored therapist‐assisted intervention sessions.

**Results:**

219 women receiving chemotherapy (*M*_age_ 50.67 years) were recruited. At baseline, average HRQoL was low (0.27). CBT‐I led to a significant improvement from baseline to post‐intervention (*b* = 0.06; *p* = 0.012). In contrast, BLT showed no significant effects (all *p* ≥ 0.519). No CBT‐I × BLT interaction was observed (all *p* ≥ 0.759). Clinically meaningful improvement (≥ MID) was observed in 71.2% of CBT‐I participants. Within‐group analyses from baseline to post‐intervention showed the largest HRQoL improvements in CBT‐I and CBT‐I + BLT groups (both b = 0.14; *p* < 0.001); smaller gains were observed in BLT and SHE groups (*b* = 0.07–0.09; *p* ≤ 0.024). No significant changes were observed in any group at 6‐month follow‐up (all *p* ≥ 0.096). Exploratory analyses suggested benefits of BLT in patients with metastatic disease and greater insomnia severity.

**Conclusion:**

CBT‐I was associated with clinically meaningful improvements in HRQoL during chemotherapy. These findings support the integration of CBT‐I into supportive care and highlight the need for tailored approaches for patients with advanced disease or persistent insomnia symptoms.

**Trial registration:**

ACTRN12620001133921

## 1 Introduction

Advances in cancer detection and treatment have improved survival; however, this does not always equate to better quality of survival. Health‐Related Quality of Life (HRQoL) is a comprehensive, patient‐reported measure that reflects how cancer and its treatment affect functioning and well‐being across physical, psychological, and social dimensions [1]. Both cancer and its treatment impact HRQoL, contributing to heightened symptom burden, reduced functioning, and increased healthcare needs, highlighting the need to identify and address factors that reduce HRQoL in cancer populations.

Sleep disturbance is a key feature of insomnia, affecting 30%–60% of cancer patients [2, 3]. This is nearly twice that of the general population, and contributes to reduced HRQoL [4, 5]. Insomnia disorder is characterized by dissatisfaction with sleep quality and/or quantity, difficulty initiating/maintaining sleep, or early morning awakenings, that occur at least three times per week for at least 3 months despite adequate opportunities for sleep, and causes daytime impairment [6]. In cancer, elevated rates of insomnia are linked to the psychological burden of diagnosis and treatment, particularly chemotherapy [3, 7], circadian rhythm disruption [8], and the direct effects of cancer therapies [9]. Interventions targeting insomnia may therefore improve HRQoL during chemotherapy.

Non‐pharmacological treatments for insomnia may improve sleep in cancer patients. Cognitive Behavioral Therapy for Insomnia (CBT‐I), the gold standard insomnia intervention, includes sleep restriction, stimulus control, relaxation, and cognitive restructuring [10]. CBT‐I reduces insomnia and outperforms pharmacological treatments [11, 12]. However, its impact on HRQoL remains unclear, due to inconsistent measurement tools (e.g., generic vs. disease‐specific, multi‐vs. single‐item), methodological variability, limited cancer‐focused research, and HRQoL's multidimensional nature [13].

Bright Light Therapy (BLT) may help regulate circadian rhythms and improve sleep during chemotherapy [14], though its impacts on HRQoL are understudied. A recent review found BLT to be a safe and potentially effective treatment for sleep, mood, and anxiety symptoms impacting HRQoL in cancer patients [15]. One pilot study reported that BLT reduced HRQoL‐related symptom worsening and improved sleep and depression [16]. Furthermore, a trial combining CBT‐I and BLT also showed improvements in insomnia and fatigue during breast cancer chemotherapy [17], suggesting potential additive benefits.

This study reports on a planned, pre‐registered, secondary analysis of the Sleep Cancer Rest (SleepCaRe) trial, HRQoL. HRQoL is important to evaluate, given its relevance to patient‐centered cancer care. Improvements in sleep do not necessarily translate to broader gains in overall HRQoL during chemotherapy, when symptom burden remains high; this secondary analysis therefore examined intervention impacts on overall HRQoL during active treatment. The primary aim of this paper was to evaluate the effects of CBT‐I and BLT on HRQoL in women with breast cancer undergoing chemotherapy. We hypothesized that [1] participants receiving CBT‐I (i.e., CBT‐I, CBT‐I + BLT) would demonstrate higher HRQoL compared to those not receiving CBT‐I (i.e., BLT, SHE), and [2] participants receiving BLT (i.e., CBT‐I + BLT, BLT) would demonstrate higher HRQoL compared to those not receiving BLT (i.e., CBT‐I, SHE).

Exploratory aims were to investigate effects of CBT‐I and BLT through 6‐month follow‐up, explore whether there is a CBT‐I × BLT interaction, and evaluate whether results are consistent across subgroups including initial insomnia severity (clinical, subthreshold), and cancer stage (metastatic).

## 2 Method

This is a planned analysis of a secondary outcome of *SleepCaRe*, a 6‐week, randomized, controlled, multi‐site, 2 × 2 factorial, superiority, parallel‐group trial registered with Australian New Zealand Clinical Trials Registry (ACTRN12620001133921). Primary outcomes include insomnia severity and fatigue symptoms. Materials (https://osf.io/msycd/) and protocol [18] are publicly available and further details are in supplement. The Human Research Ethics Committee of the Peter MacCallum Cancer Center approved the study (HREC/55622/PMCC). Reporting followed the Consolidated Standards of Reporting Trials (CONSORT) for social and psychological interventions (CONSORT‐SPI) and patient‐reported outcomes (CONSORT‐PRO) [19, 20].

### 2.1 Participants

Eligible participants were adult women with breast cancer undergoing cytotoxic chemotherapy (intravenous or oral), with at least 6 weeks of treatment remaining and regular internet access. Eligibility did not require a clinical symptom threshold, as sleep disturbance during chemotherapy is common, fluctuates over time, and often does not meet diagnostic criteria despite being clinically meaningful. This inclusive approach was intended to enhance ecological validity and reflect real‐world oncology populations. Metastatic (i.e., stage four) patients were included as evidence highlights a paucity of research on health behaviors, including sleep, in advanced cancer despite high symptom burden and unmet supportive care needs [21]. Anticipated heterogeneity was addressed through stratified randomisation and explored via pre‐specified subgroup analyses. Exclusion criteria included being male; a history of migraines or brain metastases requiring daily steroids; current or planned use of sleep medications/herbal aids; severe psychiatric or substance use disorders; and untreated sleep disorders.

### 2.2 Interventions

All interventions involved self‐directed (therapeutic and psychoeducational self‐guided emails) and therapist‐assisted components (phone/video conferencing sessions) tailored to the needs of cancer patients (Table 1). Trial therapists were clinical psychology PhD trainees or research assistants trained in sleep interventions. Clinical supervision was provided by a clinical psychologist, and therapist adherence was monitored using session checklists and by recording all sessions.

**TABLE 1:** SleepCaRe interventions.

	BLT	No BLT
CBT‐I	CBT‐I + BLT CBT‐I and BLT interventions simultaneously. 75‐min intervention session. 15‐min mid‐point phone call. 12 twice‐weekly emails.	CBT‐I Core domains [1]: behavioral strategies (e.g., stimulus control, bed restriction) [2]; cognitive strategies (addressing dysfunctional beliefs about sleep) [3]; cancer‐specific strategies (e.g., mindfulness for physical discomfort). 60‐min intervention session. 15‐min mid‐point phone call. 12 twice‐weekly emails.
No CBT‐I	BLT Daily 20‐min Luminette light glasses use at habitual wake time. Tailored to individual sleep‐wake schedules, focusing on promoting morning light exposure and evening darkness. 45‐min intervention session. 15‐min mid‐point phone call. Six weekly emails.	SHE Active control condition. General sleep education and hygiene (e.g., underlying sleep processes, environmental factors) practices without CBT‐I or BLT elements. 30‐min intervention session. 15‐min mid‐point phone call. Six weekly emails.

*Note:* All conditions received sleep hygiene education (SHE) information.

### 2.3 Procedure

Between 22‐January‐2021 and 21‐August‐2024, 219 eligible women consented and were randomized via eHealth system reports, oncology referrals, or study advertisements across five Australian tertiary hospitals. Interested individuals received an introductory message and, upon providing written or recorded verbal consent, completed a screening call. Participants were randomized to one of four groups: CBT‐I, BLT, CBT‐I + BLT, SHE. Though the study was non‐blinded, all interventions were presented as potentially beneficial for sleep and fatigue. Randomization was performed in a 1:1:1:1 ratio using a pre‐generated sequence with variable block sizes (4 or 8), stratified by site, cancer stage (≤ 3 or 4), and baseline Insomnia Severity Index (ISI; ≤ 7, ≥ 8).

### 2.4 HRQoL

HRQoL was measured using the Patient‐Reported Outcomes Measurement Information System (PROMIS) [22, 23], specifically the PROMIS‐Preference (PROPr) score [24]. Seven subdomain scores were aggregated into a single summary, anchored at 0 “Dead” and 1 “Full Health”, and ranging from −0.022 (indicating “All Worst”) to 1. Where available, cancer‐specific PROMIS item banks were utilized, including for Fatigue, Depression, Pain Interference, and Physical Function. We used the recommended PROPr minimally important difference (MID) of 0.04 [25]. However, the PROPr MID has not yet been specifically validated in oncology populations, and preference‐based measures may differ in sensitivity from commonly used cancer‐specific instruments. As such, MID‐based interpretations should be considered provisional.

### 2.5 Statistical Analysis

A detailed analysis plan is written in the *Supplementary Materials “Statistical analysis” section*. The trial was powered to detect a minimally important difference on its primary outcomes, which approximately equates to a moderate 0.5 standardized mean difference (see https://github.com/behavioralmedicinelab/SleepCarePlanning for Monte Carlo power simulations). While not specifically powered for HRQoL, the sample size should be sufficient to detect an approximately moderate or larger effect size. Analyses were performed using R‐version 4.2.1 [26] and MplusAutomation [27] on an intention‐to‐treat basis and following a pre‐registered analysis plan to use latent growth models (LGMs) with a linear slope during the intervention [18]. All models adjusted for strata variables. No outliers were identified; data were approximately normally distributed. LGMs testing differences between and within groups in repeated measures data were estimated using two linear slopes. We used full information maximum likelihood for missing data, which is common for LGMs [28], unlike repeated measures ANOVA. LGMs and mixed models are mathematically equivalent under certain conditions [28]; we chose LGMs due to software convenience specifying our exact models. Hypotheses 1 and 2, testing between group differences in HRQoL, were tested by comparing groups on *slope 1* which is the change during the intervention (i.e., baseline to post‐intervention). *Slope 2* tests the exploratory aim of change during the follow‐up period (i.e., post‐intervention to follow‐up). Slope 1 includes baseline (T^0^) to mid‐point (T^1^) to post‐intervention (T^2^) timepoints with a linear specification. Slope 2 includes post‐intervention (T^2^) to 3‐month (T^3^) and 6‐month (T^4^) follow‐up timepoints with a linear specification, separate from Slope 1. The first linear slope was part of our pre‐registered analysis plan, and was chosen as it balanced our expectations for change with a specification that allows change during intervention to be summarized with a single parameter (slope) which reduces multiple comparisons when testing the main effects of CBT‐I and BLT, versus a non‐linear specification which would have multiple parameters further expanding the number of hypothesis tests and potential for false positive or if corrected for comparisons reducing power. Although not pre‐specified, for similar reasons we opted for a linear slope during the follow‐up period. Slopes 1 and 2 are separate linear slopes, which collectively allow for a different average rate of change during the active intervention from follow‐up. A standardized mean difference (SMD) effect size was calculated based on the standard deviation of the PROPr score at baseline for the entire sample. Exploratory subgroups reporting focused on main effects across slopes 1 and 2. False Discovery Rate (FDR) corrections were applied to main effect analyses of CBT‐I and BLT for Slopes 1 and 2 only, FDR corrections were not applied to exploratory subgroup analyses.

While not a main hypothesis, we also tested the within‐group change by examining slopes 1 and 2 within each group. In addition, we conducted three exploratory analyses to examine potential drivers of change in HRQoL, as assessed by PROPr scores. These analyses aimed to identify temporal patterns of change, estimate the contribution of sleep and fatigue‐related factors, and assess the clinical meaningfulness of observed improvements.

## 3 Results

**FIGURE 1:**
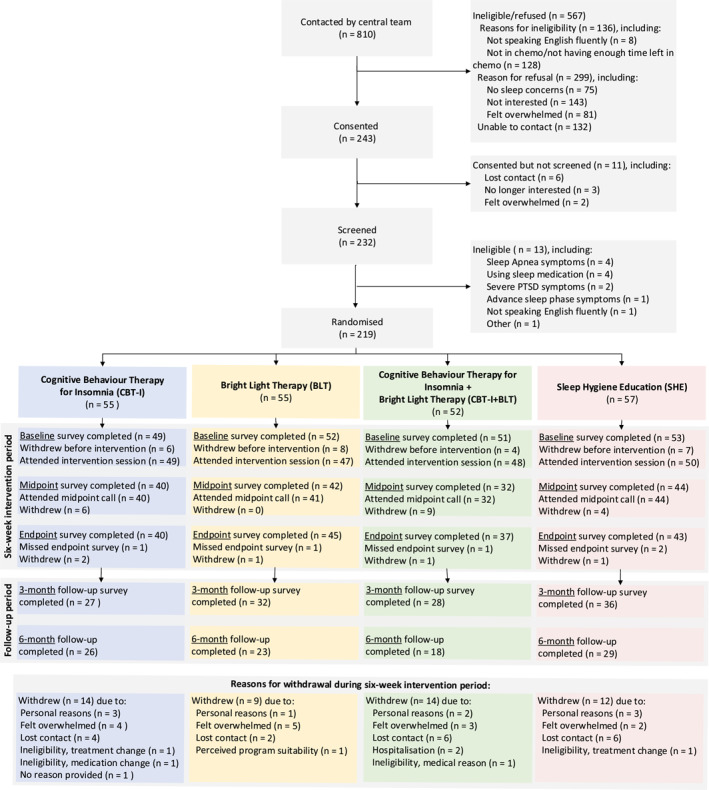
Participant flow chart.

219 women receiving cytotoxic chemotherapy for any stage of breast cancer were randomized (Figure 1). Most participants were middle‐aged (mean age 50.67 years) with early‐stage breast cancer (73.1%; Table 2 and Supporting Information S1: Table S2). The average PROPr score was 0.27. Missing data rates and reasons for withdrawal are presented in Figure 1.

**TABLE 2:** Participant demographic and clinical characteristics at baseline (T0), by intervention group.

Characteristics	CBT‐I	BLT	CBT‐I + BLT	SHE	Overall Cohort
Age, years, *M* (*SD*)	49.53 (10.49)	50.27 (11.29)	51.06 (11.44)	51.66 (10.11)	50.67 (10.77)
Education status, *n (%)*					
Less than bachelor's	13 (26.0%)	22 (41.5%)	18 (38.3%)	19 (36.5%)	72 (35.6%)
Bachelor's degree	26 (52.0%)	18 (34.0%)	14 (29.8%)	18 (34.6%)	76 (37.6%)
Higher than bachelor's	11 (22.0%)	13 (24.5%)	15 (31.9%)	15 (28.8%)	54 (26.7%)
Language spoken at home, *n* (%)					
English	42 (85.7%)	39 (73.6%)	37 (74.0%)	45 (84.9%)	163 (79.5%)
Others	7 (14.3%)	14 (26.4%)	13 (26.0%)	8 (15.1%)	42 (20.5%)
Employment Level, *n* (%)					
Employed	32 (64.0%)	38 (73.1%)	29 (59.2%)	33 (62.3%)	132 (64.7%)
Unemployed	18 (36.0%)	14 (26.9%)	20 (40.8%)	20 (37.7%)	72 (35.3%)
Marital status, *n* (%)					
Married/In a relationship	28 (57.1%)	35 (66.0%)	34 (69.4%)	32 (60.4%)	129 (63.2%)
Single/Divorced/Widowed	21 (42.9%)	18 (34.0%)	15 (30.6%)	21 (39.6%)	75 (36.8%)
Yearly income quartiles^a^, *n* (%)					
1^st^ (< $49,000)	7 (17.5%)	7 (15.6%)	9 (25.0%)	7 (15.2%)	30 (18.0%)
2^nd^ ($49,000–$99,000)	13 (32.5%)	16 (35.6%)	11 (30.6%)	19 (41.3%)	59 (35.3%)
3^rd^ ($99,000–$149,999)	5 (12.5%)	8 (17.8%)	8 (22.2%)	11 (23.9%)	32 (19.2%)
4^th^ (> $150,000)	15 (37.5%)	14 (31.1%)	8 (22.2%)	9 (19.6%)	46 (27.5%)
Location, *n* (%)					
Metropolitan	50 (90.9%)	48 (87.3%)	45 (86.5%)	46 (80.7%)	189 (86.3%)
Regional/rural/remote	5 (9.1%)	7 (12.7%)	7 (13.5%)	11 (19.3%)	30 (13.7%)
Site, *n* (%)					
Bendigo health	3 (5.5%)	5 (9.1%)	3 (5.8%)	6 (10.5%)	17 (7.8%)
Grampians health	1 (1.8%)	0 (0.0%)	1 (1.9%)	1 (1.8%)	3 (1.4%)
Mater hospital	9 (16.4%)	5 (9.1%)	3 (5.8%)	5 (8.8%)	22 (10.0%)
Monash health	8 (14.5%)	8 (14.5%)	10 (19.2%)	7 (12.3%)	33 (15.1%)
Peter MacCallum cancer center	34 (61.8%)	37 (67.3%)	35 (67.3%)	38 (66.7%)	144 (65.8%)
PROPr, M (SD)	0.25 (0.15)	0.29 (0.19)	0.27 (0.21)	0.26 (0.14)	0.27 (0.17)
Months since diagnosis, MDN (IQR)	3.50 (6.12)	3.22 (5.86)	2.79 (3.20)	2.99 (2.21)	3.19 (3.71)
Cancer stage, *n* (%)					
1	5 (10.2%)	7 (13.5%)	10 (19.6%)	7 (14.3%)	29 (14.4%)
2	17 (34.7%)	19 (36.5%)	15 (29.4%)	10 (20.4%)	61 (30.3%)
3	14 (28.6%)	12 (23.1%)	14 (27.5%)	17 (34.7%)	57 (28.4%)
4 (metastatic)	13 (26.5%)	14 (26.9%)	12 (23.5%)	15 (30.6%)	54 (26.9%)
Menopause prior to diagnosis, *n* (%)					
No	31 (56.4%)	37 (67.3%)	27 (51.9%)	29 (50.9%)	124 (56.6%)
Yes	17 (30.9%)	16 (29.1%)	23 (44.2%)	22 (38.6%)	78 (35.6%)
Unknown	7 (12.7%)	2 (3.6%)	2 (3.8%)	6 (10.5%)	17 (7.8%)
Surgery since diagnosis, *n* (%)					
No	23 (46.0%)	24 (45.3%)	24 (47.1%)	26 (49.1%)	97 (46.9%)
Yes	27 (54.0%)	29 (54.7%)	27 (52.9%)	27 (50.9%)	110 (53.1%)

*Note:* For additional Participant Demographic and Clinical Characteristics information, see Supporting Information S1: Table S2. Missing income data may be due to participant preference to not have information reported, via selection of the option “prefer not to say”. Percentages are of available data; denominators vary by variable due to missing responses.

Abbreviations: IQR, interquartile range; M, mean; MDN, median, SD, standard deviation.

^a^ = Income categories based on 2021 Australia Household Income Quartiles (see Supporting Information S1: Table S3).

### 3.1 Between‐Groups (Hypotheses 1 and 2)

**FIGURE 2:**
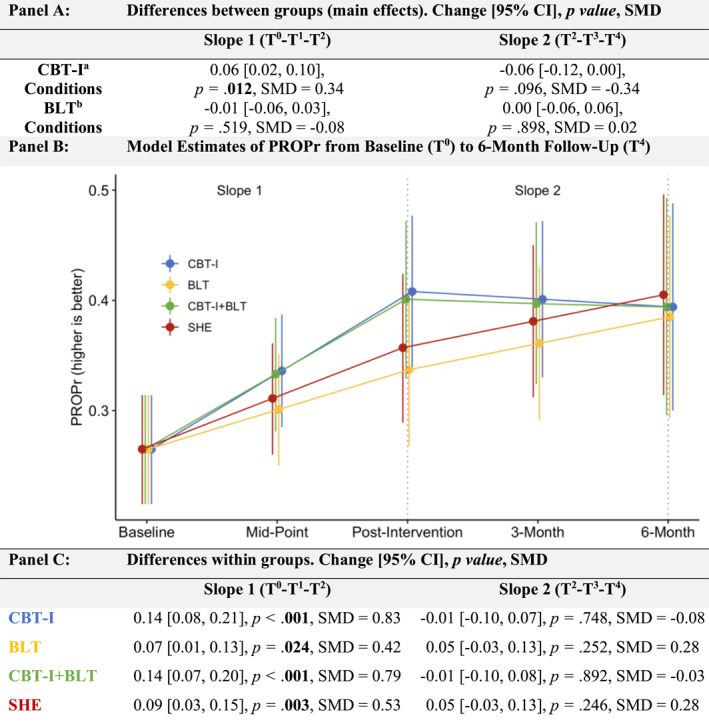
Within and between‐group effects on HRQoL (PROPr). Slope 1 includes baseline (T^0^) to mid‐point (T^1^) to post‐intervention (T^2^) timepoints. Slope 2 includes post‐intervention (T^2^) to 3‐month (T^3^) and 6‐month (T^4^) follow‐up timepoints. SMD = Standardized Mean Difference. SMD can be interpreted comparative to Cohen's d: small (SMD = 0.2), medium (SMD = 0.5), and large (SMD ≥ 0.8). Main effect analysis *p* values were adjusted for multiple comparisons using the false discovery rate (see Methods). ^a^ = Average effects of all conditions with CBT‐I versus the average effects of all conditions without CBT‐I. ^b^ = Average effects of all conditions with BLT versus the average effects of all conditions without BLT.

Our overall model had acceptable model fit indices (Supporting Information S1: Table S1). Figure 2 shows the main effects, plots the adjusted, model estimated means by group, and the within‐group changes across Slopes 1 and 2. Main effects contrasted conditions with versus without CBT‐I (i.e., CBT‐I, CBT‐I + BLT vs. BLT, SHE) and with versus without BLT (i.e., CBT‐I + BLT, BLT vs. CBT‐I, SHE; Figure 2 Panel A). The results showed a significant, main effect of CBT‐I on Slope 1 with CBT‐I groups improving an average of 0.06 HRQoL points more than groups without CBT‐I (small‐to‐moderate effect size; 95% CI [0.01, 0.10]; *p =* 0.012; SMD = 0.34). 71.2% of participants receiving CBT‐I (CBT‐I or CBT‐I + BLT) achieved ≥ the MID, compared to 50.6% of participants not receiving CBT‐I (BLT or SHE; Supporting Information S1: Table S4 Panel C). There was no significant group difference with an average difference of −0.06 points during follow‐up on Slope 2 (95% CI [−0.12, 0.00]; *p =* 0.096, SMD = −0.34). These main effects can be seen visually in Figure 2 Panel B. There was no main effect of BLT on either Slope 1 (average difference of −0.01 [95% CI [−0.06, 0.03]; *p =* 0.519; SMD = −0.08) nor Slope 2 average difference of 0.00 95% CI [−0.06, 0.06]; *p =* 0.989; SMD = 0.02 (Figure 2 Panels A and B) which showed negligible effect sizes, with 55.8% of participants who received BLT (CBT‐I + BLT, BLT) reaching ≥ the MID than those who did not (63.9%; CBT‐I, SHE; Supporting Information S1: Table S4 Panel C).

Raw means by intervention group over time are graphed in Supporting Information S1: Figure S1. The exploratory test of the CBT‐I × BLT interaction showed no significant interactions for either baseline to post‐intervention (referred to hereafter as Slope 1; *p =* 0.759) nor from post‐intervention to follow‐up (referred to hereafter as Slope 2; *p* = 0.898). This supports our reporting focused on the hypothesized main effects of CBT‐I and BLT.

### 3.2 Exploratory Analyses

#### 3.2.1 Within‐Groups

Within‐group effects assessed changes in each of the four conditions. Results showed a significant within‐group improvement in HRQoL in all conditions for Slope 1 (Figure 2 Panel C), with the largest improvements of 0.14 HRQoL points for both the CBT‐I (*p* < 0.001; SMD = 0.83) and CBT‐I + BLT groups (*p* < 0.001; SMD = 0.79), and smaller improvements of 0.09 points in SHE (*p* = 0.003; SMD = 0.53) and 0.07 points in BLT groups (*p* = 0.024; SMD = 0.42). For Slope 2, small non‐significant improvements were observed across the conditions (all *p ≥* 0.246). Raw, individual participant change scores across Slope 1 and Slope 2 are in Supporting Information S1: Figure S2 revealing a majority of participants with improved HRQoL.

#### 3.2.2 Drivers of Change

We examined changes in subdomains to explore drivers of change in HRQoL; descriptive change scores suggested that most symptom improvements occurred during the intervention period (T^2^‐T^0^), particularly in sleep and fatigue‐related domains (Supporting Information S1: Table S4; Panel A). Supporting these change scores, linear regression models showed that changes in sleep and fatigue‐related symptoms (sleep disturbance, fatigue, insomnia severity, and sleep‐related impairment) explained a substantial proportion of variance in HRQoL improvements across timepoints, ranging from 55.9% to 60.5% (Supporting Information S1: Table S4; Panel B). These findings underscore the central role of sleep and fatigue in driving overall HRQoL gains.

#### 3.2.3 Subgroup Analyses: Stage, Insomnia Severity

No significant CBT‐I × BLT interactions were observed across all subgroup analyses, including cancer stage (Slope 1: *p* = 0.167, Slope 2: *p* = 0.829), clinical insomnia severity (Slope 1: *p* = 0.072, Slope 2: *p* = 0.403), and subthreshold insomnia severity (Slope 1: *p* = 0.298, Slope 2: *p* = 0.398); therefore, main effects were examined (Table 3, Panels A, C, and E, respectively). Subgroup sample sizes are reported in Table 3 (metastatic disease: *n* = 51; clinical insomnia severity: *n* = 84; subthreshold insomnia severity: *n* = 123). All subgroup analyses were exploratory and not powered to detect subgroup‐specific effects.

**TABLE 3:** Subgroup analyses: Within and between‐group effects on overall HRQoL (PROPr).

Cancer stage (metastatic, stage 4) subgroup^c^ (overall subgroup *n* = 51)		
PROPr	Slope 1 (T^0^‐T^1^‐T^2^)	Slope 2 (T^2^‐T^3^‐T^4^)
Panel A: Differences between groups (main effects). Change [95% CI], *p* value, SMD		
CBT‐I^a^ conditions	0.05 [−0.03, 0.13], *p =* 0.210, SMD = 0.30	0.03 [−0.08, 0.13], *p =* 0.620, SMD = 0.15
BLT^b^ conditions	0.06 [−0.02, 0.14], *p =* 0.210, SMD = 0.36	0.04 [−0.06, 0.14], *p =* 0.620, SMD = 0.25
Panel B: Differences within groups (simple effects). Change [95% CI], *p* value, SMD		
CBT‐I	0.16 [0.04, 0.29], *p =* 0.013, SMD = 0.95	−0.08 [−0.24, 0.07], *p =* 0.290, SMD = −0.48
BLT	0.18 [0.05, 0.30], *p =* 0.008, SMD = 1.01	−0.07 [−0.22, 0.09], *p =* 0.408, SMD = −0.38
CBT‐I + BLT	0.28 [0.15, 0.42], *p <* 0.001, SMD = 1.64	−0.03 [−0.20, 0.14], *p =* 0.743, SMD = −0.16
SHE	0.17 [0.04, 0.30], *p =* 0.012, SMD = 0.97	−0.10 [−0.25, 0.05], *p* = 0.204, SMD = −0.57

**Clinical** **insomnia** **severity (ISI ≥ 15)** **subgroup** **^d^** **(overall subgroup** ***n* = 84)**		
PROPr	Slope 1 (T^0^‐T^1^‐T^2^)	Slope 2 (T^2^‐T^3^‐T^4^)
Panel C: Differences between groups (main effects). Change [95% CI], *p* value, SMD		
CBT‐I^a^ conditions	0.09 [0.02, 0.15], *p =* 0.014, SMD = 0.51	−0.09 [−0.19, 0.00], *p =* 0.110, SMD = −0.53
BLT^b^ conditions	0.04 [−0.03, 0.10], *p =* 0.285, SMD = 0.21	−0.00 [−0.09, 0.09], *p =* 0.981, SMD = 0.01
Panel D: Differences within groups (simple effects). Change [95% CI], *p* value, SMD		
CBT‐I	0.11 [0.01, 0.20], *p =* 0.034, SMD = 0.61	0.05 [−0.09, 0.18], *p =* 0.477, SMD = −0.53
BLT	0.05 [−0.04, 0.15], *p =* 0.288, SMD = 0.30	0.14 [−0.00, 0.29], *p =* 0.053, SMD = 0.82
CBT‐I + BLT	0.20 [0.10, 0.30], *p <* 0.001, SMD = 1.16	0.01 [−0.13, 0.15], *p =* 0.888, SMD = 0.06
SHE	0.08 [−0.02, 0.17], *p =* 0.115, SMD = 0.45	0.10 [−0.03, 0.23], *p =* 0.130, SMD = 0.58

**Subthreshold** **insomnia** **severity (ISI ≤ 14)** **subgroup** **^e^** **(overall subgroup** ***n* = 123)**		
PROPr	Slope 1 (T^0^‐T^1^‐T^2^)	Slope 2 (T^2^‐T^3^‐T^4^)
Panel E: Differences between groups (main effects). Change [95% CI], *p* value, SMD		
CBT‐I^a^ conditions	0.04 [−0.02, 0.09], *p =* 0.212, SMD = 0.20	−0.06 [−0.13, 0.02], *p =* 0.294, SMD = −0.32
BLT^b^ conditions	−0.04 [−0.10, 0.01], *p =* 0.212, SMD = −0.24	0.03 [−0.05, 0.10], *p =* 0.471, SMD = 0.16
Panel F: Differences within groups (simple effects). Change [95% CI], *p* value, SMD		
CBT‐I	0.17 [0.08, 0.25], *p <* 0.001, SMD = 0.95	−0.09 [−0.20, 0.02], *p =* 0.108, SMD = −0.52
BLT	0.09 [0.01, 0.18], *p =* 0.037, SMD = 0.52	0.01 [−0.11, 0.10], *p =* 0.877, SMD = −0.05
CBT‐I + BLT	0.10 [0.01, 0.19], *p* = 0.038, SMD = 0.56	−0.03 [−0.15, 0.09], *p =* 0.614, SMD = −0.18
SHE	0.10 [0.02, 0.19], *p =* 0.016, SMD = 0.59	0.00 [−0.11, 0.10], *p =* 0.944, SMD = −0.02

*Note:* Slope 1 includes baseline (T^0^) to mid‐point (T^1^) to post‐intervention (T^2^) timepoints. Slope 2 includes post‐intervention (T^2^) to 3‐month (T^3^) and 6‐month (T^4^) follow‐up timepoints. SMD = Standardized Mean Difference. SMD can be interpreted comparative to Cohen's d: small (SMD = 0.2), medium (SMD = 0.5), and large (SMD ≥ 0.8). Main effect analysis *p* values were adjusted for multiple comparisons using the false discovery rate (see Methods). Missing data may account for subgroup sizes not computing to overall subgroup total.

^a^ = Average effects of all conditions with CBT‐I versus the average effects of all conditions without CBT‐I.

^b^ = Average effects of all conditions with BLT versus the average effects of all conditions without BLT.

^c^ = Metastatic cancer subgroup includes all participants with stage 4 cancer.

^d^ = Clinical insomnia symptom severity subgroup includes all participants with an Insomnia Severity Index (ISI) score of > 15 on the ISI measure (i.e., clinical insomnia—moderate to severe severity) at screening (see Methods).

^e^ = Subthreshold insomnia symptom severity subgroup includes all participants with an Insomnia Severity Index (ISI) score of ≤ 14 on the ISI measure (i.e., subthreshold insomnia) at screening (see Methods).

#### 3.2.4 Cancer Stage (Metastatic)

Neither CBT‐I nor BLT significantly influenced Slope 1 or Slope 2 (*p* ≥ 0.210). However, both interventions showed small‐to‐moderate effect sizes in Slope 1, with BLT (0.06 point‐change; SMD = 0.36) yielding a slightly larger improvement in HRQoL than CBT‐I (0.05 point‐change; SMD = 0.30). Similarly, in Slope 2 BLT demonstrated a larger effect size (0.04 point‐change; SMD = 0.25) than CBT‐I (0.03 point‐change; SMD = 0.15).

#### 3.2.5 Clinical Insomnia Severity (ISI > 15)

CBT‐I significantly improved HRQoL in Slope 1 (0.09 point‐change; *p* = 0.014, SMD = 0.51), to moderate effect. While no other significant main effects were observed (all *p* ≥ 0.110), BLT showed a small, non‐significant HRQoL improvement in Slope 1 (0.04 point‐change; SMD = 0.21). In Slope 2, CBT‐I exhibited a moderate decline (−0.09 point‐change; SMD = −0.53), suggesting a potential plateau over the follow‐up, while BLT had no meaningful change (no change; SMD = 0.01).

#### 3.2.6 Subthreshold Insomnia Severity (ISI ≤ 14)

Neither CBT‐I nor BLT had significant effects on HRQoL across timepoints. However, CBT‐I showed a small, non‐significant HRQoL increase in Slope 1 (0.04 point‐change; SMD = 0.20), whereas BLT exhibited a small decline (−0.04 point‐change; SMD = −0.24). In Slope 2, CBT‐I was associated with a small decline in HRQoL (−0.09 point‐change; SMD = −0.32) while BLT showed a slight improvement in HRQoL (0.03 point‐change; SMD = 0.16).

## 4 Discussion

### 4.1 Overall HRQoL

#### 4.1.1 Between‐Groups (Main Effects)

CBT‐I significantly improved HRQoL during chemotherapy, extending prior findings in post‐treatment cancer survivors to those undergoing chemotherapy. These improvements were moderate in size and maintained at follow‐up, though gains plateaued, a pattern consistent with earlier research [29]. This plateau, expected due to typical HRQoL declines during chemotherapy, may indicate the intervention's maximum benefit was reached or point to the utility of booster sessions for patients experiencing ongoing side effects [30]. CBT‐I may influence HRQoL through broader psychological and behavioral mechanisms, including enhanced self‐efficacy, reduced maladaptive illness‐related cognitions, improved emotion regulation, and greater perceived control during treatment. CBT‐I's structured, skills‐based approach may also promote adaptive coping and behavioral activation, which are closely linked to HRQoL domains such as emotional well‐being and social functioning.

Importantly, a substantially higher proportion of participants in the CBT‐I groups achieved ≥ the MID, compared to those in non‐CBT‐I groups. This ∼20% difference underscores CBT‐I's added value, not only in statistical terms but in real‐world, patient‐perceived benefit. In contrast, no comparable pattern was observed for participants who received BLT versus those who did not, suggesting that BLT may offer limited standalone benefits for HRQoL during chemotherapy.

In contrast, BLT did not significantly impact HRQoL. This may reflect insufficient circadian disruption within the sample, or limited understanding of BLT's underlying mechanisms in cancer contexts [10, 31]. BLT was delivered as a relatively light‐touch, morning‐based intervention, which may be insufficient to induce meaningful circadian phase shifts during chemotherapy, and undetectable without specific biological markers of circadian phase, particularly if circadian misalignment was mild or heterogenous [32, 33]. It is also possible that the strength of circadian signal exposure, rather than timing alone, is critical for downstream HRQoL effects. Thus, BLT may be more effective when delivered as a more intensive lighting intervention or when targeted to individuals with pronounced circadian delay.

The absence of a significant CBT‐I and BLT may be a due to a lack of power or that they do not exert synergistic or antagonistic effects on HRQoL when combined. While both have been shown to improve sleep individually [17, 29, 34], their joint delivery may not yield additional HRQoL benefits beyond CBT‐I alone.

#### 4.1.2 Within‐Groups

All four conditions showed HRQoL improvements during treatment, with the largest changes observed in the CBT‐I and CBT‐I + BLT groups. These align with previous findings that CBT‐I improves sleep and yields secondary HRQoL benefits [35, 36]. CBT‐I + BLT did not outperform CBT‐I alone, highlighting the primary role of behavioral and cognitive strategies. In contrast, BLT and SHE produced smaller, less consistent effects. Given the use of an active control and the provision of sleep hygiene content across all conditions, improvements observed across groups may reflect a combination of non‐specific intervention effects, increased symptom awareness, expectancy effects, and natural symptom fluctuations during chemotherapy. Without a treatment‐as‐usual or waitlist control, it remains difficult to fully disentangle treatment‐specific effects from these broader influences.

Importantly, the magnitude of within‐group improvement observed in the SHE condition was larger than anticipated for an active control. This may reflect the clinical relevance of basic sleep education during chemotherapy, where even modest behavioral changes can reduce symptom burden. Improvements may also reflect non‐specific factors such as increased symptom awareness, expectancy effects, or therapeutic rapport resulting from active therapist involvement.

Finally, across all conditions, gains were maintained at follow‐up, though they did not continue accumulating; a plateau that may relate to the natural conclusion of chemotherapy cycles (∼6–12 weeks), which overlapped with the study's follow‐up window.

Notably, symptom trajectories suggest that most HRQoL gains occurred during the active intervention phase, particularly among those receiving CBT‐I. Sleep and fatigue improvements emerged early and were among the most pronounced, suggesting they may serve as proximal mechanisms driving downstream improvements in overall HRQoL. This timing is consistent with the structure of CBT‐I, which targets these domains directly and early in treatment.

#### 4.1.3 Exploratory Subgroup Analyses

Given the exploratory nature of below subgroup findings, results are intended to be interpreted as hypothesis‐generating rather than confirmatory.

##### 4.1.3.1 Cancer Stage (Metastatic, Stage 4)

In participants with metastatic cancer (26.9% sample), both CBT‐I and BLT showed HRQoL improvements at follow‐up, comparable to CBT‐I's effects in the overall sample. The sustained gains suggest potential long‐term benefit, with BLT showing a larger effect size than CBT‐I in this subgroup. This may support BLT's use as a low‐burden therapy for advanced cancer patients. The relatively smaller effect of CBT‐I may relate to more severe or persistent treatment‐related sleep disturbances. However, although observed trajectories may suggest potential intervention benefit, these effects require confirmation in larger, specifically powered trials.

##### 4.1.3.2 Clinical Insomnia Severity

There was a higher magnitude of change among participants with moderate‐to‐severe insomnia (ISI > 15, 38% of the sample), where CBT‐I led to greater HRQoL improvements during treatment than in the overall sample. However, gains plateaued at follow‐up, indicating a potential need for ongoing support (e.g., booster sessions), or that HRQoL may naturally recover over time, with interventions expediting early improvements (e.g., within ∼6 weeks vs. ∼6 months). Participants with subthreshold insomnia (ISI ≤ 14, 56% of the sample), CBT‐I had smaller, non‐significant effects on HRQoL, suggesting that CBT‐I and BLT may be less effective in individuals with milder sleep symptoms.

### 4.2 Strengths, Limitations, and Future Directions

This study has several strengths. It was a multi‐centre, randomized clinical trial with longitudinal follow‐up at three and 6 months. The use of the PROPr system enabled patient‐centered HRQoL measurement by capturing individual preferences and avoiding ceiling and floor effects common in other tools [24]. An active control (SHE) allowed the isolation of treatment‐specific effects from non‐specific therapeutic factors (e.g., expectations, attention). While SHE provides basic sleep education, it is not considered an efficacious standalone treatment [37], thus offering an appropriate comparator.

There are several limitations. First, the study was not powered to detect small‐to‐moderate effects on HRQoL, which may be indirect and influenced by multiple mediating variables (e.g., mood, symptom burden). Our exploratory subgroup, interaction, and subdomain analyses are not corrected for multiple comparisons so should be viewed as exploratory and hypothesis‐generating requiring replication and confirmation in future studies. The sample was also limited to English‐speaking women with breast cancer, restricting generalizability to other genders, cancer types, and culturally and linguistically diverse populations. In addition, it is possible that participants who were less burdened by treatment or associated costs (e.g., financial toxicity, cultural or linguistic barriers) were more likely to enroll, which may further limit the representativeness of the sample. Moreover, 23 out of 49 participants who withdrew, many citing feeling overwhelmed or unwell/personal reasons, highlighting treatment burden as a barrier to engagement, particularly in regional settings. Withdrawal may have been influenced by COVID‐19 pandemic‐related stressors, particularly in regions like Melbourne with extended lockdowns. In addition, expectancy effects may have contributed to improvements, given active therapist involvement across conditions. Further to this, heterogeneity in cancer stage and chemotherapy regimens may have increased variability in HRQoL trajectories, potentially attenuating between‐group differences. Finally, exploratory subgroup, interaction, and subdomain analyses are not corrected for multiple comparisons so should be viewed as exploratory and hypothesis generating requiring replication and confirmation in future studies.

Future research should focus on strategies to improve retention and explore alternative or adjunctive treatments for individuals with more severe symptomologies that may hinder engagement. For instance, through stepped‐care models, which balance treatment burden and benefit by providing more intensive interventions only when necessary. Comparison against a treatment‐as‐usual or waitlist control would be worthwhile to better isolate potential naturalistic improvements. While the present study focused on sleep interventions during active chemotherapy, sleep disturbances often persist into survivorship and may be compounded by factors such as fear of cancer recurrence, treatment‐induced menopause, and ongoing endocrine therapy [38]. These challenges may contribute to the plateau effect observed in some interventions. Given that emotion‐ and lifestyle‐focused interventions have shown limited efficacy in improving sleep during re‐entry survivorship [39], future work should assess the current intervention's effectiveness beyond treatment completion. Additionally, as chemotherapy may disrupt circadian rhythms through poorly understood mechanisms [14], research should examine optimization of BLT parameters like duration, frequency, and timing relative to chemotherapy cycles and sleep‐wake disorder symptomology. Furthermore, a recent review exploring BLT in oncological populations highlighted the need for HRQoL‐focused research [15]. This emphasizes the importance of further exploration of BLT's potential effects on HRQoL in cancer. Lastly, future research is needed to establish cancer‐specific MIDs for preference‐based HRQoL measures such as PROPr.

### 4.3 Implications of Findings

This study provides evidence that CBT‐I can significantly improve HRQoL in breast cancer patients undergoing chemotherapy. Given that HRQoL often declines during chemotherapy, these early improvements are clinically meaningful.

While CBT‐I + BLT did not outperform CBT‐I alone, both demonstrated benefits. BLT and SHE showed modest effects and may serve as low‐burden adjunctive options or starting points in a stepped‐care model.

Importantly, the findings suggest that integrating CBT‐I into supportive care could be most appropriate for patients with clinically significant insomnia (e.g., ISI > 15). For those with milder symptoms (e.g., ISI ≤ 14), lower‐intensity options such as SHE may suffice. This tiered approach balances effectiveness and availability, particularly in resource‐limited settings or areas where patients have limited access to psychological care, such as remote and regional patients (13.7% of sample).

The online format used in this trial reduced barriers such as travel and financial strain, allowing participants to engage in treatment while undergoing chemotherapy. Compared to prior trials [29], which did not report on geographic accessibility, our study contributes important evidence by addressing this gap by highlighting the scalability and equity of internet‐delivered interventions. Future implementation efforts should focus on refining telehealth‐based interventions for cancer populations, ensuring accessibility, and targeting delivery based on insomnia severity.

## 5 Conclusion

This study highlights the importance of addressing sleep disturbances during chemotherapy. CBT‐I significantly improved HRQoL in breast cancer patients, with the largest effects observed during the intervention period with promising maintenance through to 6‐months follow‐up. Indeed, 71.2% of individuals who received CBT‐I (with or without BLT), compared to 50.6% of participants not receiving CBT‐I, had clinically meaningful improvements in HRQoL. BLT and SHE may offer smaller gains and could be integrated as lower‐intensity or adjunctive options. By using an online format, this research underscores the potential for scalable, accessible treatment options that address geographic and systemic barriers to care. Future research should explore targeted implementation strategies (e.g., stepped care or screening‐based triage) to optimize resource use and patient outcomes and ensure broader accessibility across diverse patient populations. Ultimately, these findings support a shift toward more personalized, patient‐centered approaches in oncology care, prioritizing the *quality* of survival alongside disease management.

## Author Contributions

**Rebecca Wallace:** conceptualization, data curation, formal analysis, investigation, methodology, project administration, visualization, writing – original draft, writing – review and editing. **Marliese Alexander:** supervision, writing – review and editing. **Daphne Day:** supervision, writing – review and editing. **Justine Diggens:** supervision, writing – review and editing. **Maria Ftanou:** supervision, writing – review and editing. **Veronica Aedo‐Lopez:** writing – review and editing. **Bei Bei:** writing – review and editing. **Robert Blum:** writing – review and editing. **Frances Boyle:** writing – review and editing. **Trang Thuy Do:** formal analysis, writing – review and editing. **Prudence A. Francis:** writing – review and editing. **Sheila N. Garland:** writing – review and editing. **Jordan Maccora:** writing – review and editing. **Sharad Sharma:** writing – review and editing. **Lesley Stafford:** writing – review and editing. **Michelle White:** writing – review and editing. **Duncan Mortimer:** formal analysis, writing – review and editing. **Joshua F. Wiley:** conceptualization, data curation, formal analysis, funding acquisition, investigation, methodology, project administration, resources, software, supervision, visualization, writing – review and editing.

## Funding

Dr. Wiley was supported by a National Health and Medical Research Council Investigator Grant (APP1178487). Frances Boyle is supported by The Friends of the Mater Foundation.

## Conflicts of Interest

The authors declare no conflicts of interest.

## Supporting information


Supporting Information S1

## Data Availability

The data that support the findings of this study are openly available in Open Science Framework at https://osf.io/msycd/overview, reference number 10.17605/OSF.IO/MSYCD.
