# Supplementary material for: A Randomized Controlled Trial: Evaluating the Sleep, Cancer and Rest (SleepCaRe) Trial to Improve Health‐Related Quality of Life in Women Undergoing Chemotherapy for Breast Cancer
Source: Psychooncology. 2026 Mar 22;35(3):e70418. doi: 10.1002/pon.70418 (PMC13006194; doi:10.1002/pon.70418)
Supplement: Supplementary file 1 — Supporting Information S1 [file PON-35-e70418-s001.docx]

Supplementary Material

Table of Contents

[Supplementary Material 1](#_Toc218069296)

[Method 3](#_Toc218069297)

[Participants – Extended Inclusion and Exclusion Criteria. 3](#_Toc218069298)

[***Inclusion Criteria.*** 3](#_Toc218069299)

[***Exclusion Criteria.*** 3](#_Toc218069300)

[***Psychopathology assessment – exclusion criteria (b).*** 4](#_Toc218069301)

[HRQoL 6](#_Toc218069302)

[Procedure 7](#_Toc218069303)

[Statistical analysis 8](#_Toc218069304)

[Statistical analysis – drivers of change exploratory analyses 10](#_Toc218069305)

[***A. Change Score Calculation*** 10](#_Toc218069306)

[***B. Variance in HRQoL Change Explained by Sleep and Fatigue Variables*** 10](#_Toc218069307)

[***C. Proportion of Participants Achieving Clinically Meaningful Change (MID Analysis)*** 11](#_Toc218069308)

[Results 12](#_Toc218069309)

[Model Information of Latent Growth Models 12](#_Toc218069310)

[Table S1. Model Fit Indices 12](#_Toc218069311)

[Participant demographics and clinical characteristics at baseline 13](#_Toc218069312)

[Table S2. Additional Participant Demographic and Clinical Characteristics at Baseline (T0), by Intervention Group 13](#_Toc218069313)

[Household income quartiles 14](#_Toc218069314)

[Table S3. Income Quartiles used in SleepCaRe 14](#_Toc218069315)

[Drivers of Change Exploratory Analyses 15](#_Toc218069316)

[Table S4. Exploratory Analyses: Change Scores, Sleep-Related Variance, and Clinical Meaningfulness 15](#_Toc218069317)

[***Key Takeaways:*** 16](#_Toc218069318)

[***Additional PROPr Figures*** 18](#_Toc218069319)

[Figure S1. Observed HRQoL Over Time from Baseline to 6 Month Follow-up 18](#_Toc218069320)

[Figure S2. Individual Participant Change in HRQoL by Condition 18](#_Toc218069321)

[Figure S3. Diagram of Latent Growth Model 19](#_Toc218069322)

[References 20](#_Toc218069323)

**Method**

**Participants – Extended Inclusion and Exclusion Criteria.**

***Inclusion Criteria.***

1. Breast cancer diagnosis;
2. Age ≥ 18 years;
3. Receiving oral or intravenous cytotoxic-based systemic therapies (e.g., chemotherapy), with at least 6 more weeks of treatment anticipated at the time of enrolment;
4. Able to understand intervention materials in English and complete surveys in English;
5. Able to provide informed consent;
6. Having regular access to email and internet.

***Exclusion Criteria.***

1. History of suffering migraines;
2. Report severe current psychopathology, including Manic and Hypomanic Episodes, Post-traumatic Stress Disorder other than cancer-related and Psychotic Disorders; OR psychopathology within the past 12 months including alcohol Dependence/Abuse and Substance Dependence/Abuse;
3. Male;
4. Daily use of sleep medications or herbal sleep aids for the previous two weeks and/or ongoing or planned daily use during the trial (occasional use is allowed)
5. Brain metastasis treated with daily steroid use;
6. Participants who show the following symptoms of sleep disorders:
   1. Sleep apnoea: loud snoring OR observed gasping or pauses in breathing OR previously diagnosed with apnoea hypopnea index >15 but not/inadequately treated, as assessed through the SCISD-R interview.
   2. Previously diagnosed Periodic Limb Movement Disorder with arousal index > 15
   3. Restless Legs Syndrome (RLS; based on the Structured Clinical Interview for Sleep Disorders (SCISD-R)) occurring ≥3 times/week, with duration of at least one month.
   4. Circadian rhythm disorders (based on the SCISD-R):

- Irregular Sleep Wake Disorder; Non-24-Hour Sleep-Wake Syndrome; Advance Sleep-Phase Syndrome (if habitual bedtime is earlier than 9 pm and habitual wake time is earlier than 5 am. Occasional deviation from this schedule is allowed.); Delayed Sleep-Phase Syndrome (if habitual bedtime is later than 2 am and habitual wake time is later than 10 am. Occasional deviation from this schedule is allowed.); Fixed night shift work between midnight and 5 a.m., or rotating work schedules that require night shifts during their participation.; Narcolepsy.
  1. Other previously diagnosed sleep disorders – if severe (discuss with CPI).

***Psychopathology assessment – exclusion criteria (b).***

Two measures were used to assess the presence of current or historical severe psychiatric and/or substance use disorder (exclusion criteria b):

- - - 1. Mini International Neuropsychiatric Interview 7.0 (MINI) (Sheehan et al., 1998)

A structured diagnostic interview designed to assess a wide range of psychiatric disorders. It is compatible with both DSM and ICD criteria and primarily focuses on current diagnoses, only exploring lifetime diagnoses when relevant. The interview uses a modular approach, with each module taking only a few minutes to administer, making it suitable for clinical settings. The MINI has demonstrated good reliability and validity across most diagnoses, although it has shown limitations in certain areas, such as generalized anxiety disorder and agoraphobia. Despite its brevity, the MINI effectively provides reliable diagnoses, contributing to its widespread use in both clinical practice and research.

- - - 1. DSM-5-TR Level 1 Cross-Cutting Symptom Measure (Clarke & Kuhl, 2014)

A self-report tool developed to assess psychiatric symptoms across multiple domains, regardless of specific diagnoses. It includes 23 questions that evaluate 13 different areas, such as depression, anxiety, substance use, and psychosis, based on the individual's experiences over the past two weeks. This measure is designed to inform clinical evaluations and track symptom changes over time, making it a versatile tool in both clinical and research settings. The DSM-5-TR measure has been well-received by clinicians and patients for its usefulness and reliability, and it allows for a more holistic view of a patient's mental health status.

During the trial, we initially used the MINI due to its comprehensive assessment capabilities. However, based on participant feedback and concerns about the high burden of completing the structured interview, we transitioned to the DSM-5-TR Level 1 Cross-Cutting Symptom Measure. Both tools are effective in assessing psychiatric and substance use disorders, but the DSM-5-TR Level 1 Cross-Cutting Symptom Measure was preferred for its shorter administration time and ease of use, which helped reduce participant fatigue while still providing valuable diagnostic information. This change was made to improve participant experience without compromising the quality of the data collected.

**HRQoL**

The PROPr score (Dewitt et al., 2018, 2020; Hanmer et al., 2018) encompassed seven distinct subdomains over the preceding week, with all items rated on a 5- point Likert-type scale: (1) Cognitive Function (e.g., “My thinking has been foggy”); (2) Physical Function (e.g., “Are you able to stand for one hour?”); (3) Pain Interference (e.g., “How often did pain make you feel discouraged?”); (4) Fatigue (e.g., “How exhausted were you on average?”); (5) Ability to Participate in Social Roles and Activities (e.g., “I have trouble meeting the needs of my family”); (6) Depression (e.g., “I felt helpless”); and (7) Sleep Disturbance (e.g., “My sleep was restful”).

States with PROPr scores less than zero (minimum score of -0.022) are possible and considered “All Worst”.

The PROPr measure was specifically designed to reduce ceiling and floor effects commonly seen in other HRQoL measures, demonstrating strong construct validity, reliability, and precision as a generic preference-based summary scoring system for HRQoL. HRQoL scores for each subdomain were collected and calculated using Computer Adaptive Testing (CAT), which dynamically tailored item selection to each participant's responses. PROMIS CAT scales have variable item administration, the CAT algorithm enhances precision by selecting the optimal number and type of items. The algorithm typically administers between 4 and 12 items, concluding when the T-score standard error is below 3.0, to ensure reliability or 12 items have been administered.

**Procedure**

The randomisation sequence was generated in REDCap by research staff who were not involved in recruitment or intervention delivery. REDCap, hosted by Monash University, is an open-source data-management tool with features such as data encryption and role-based security. An authorized staff member entered the eligibility and stratification data in REDCap to obtain group allocation.

Participants were subsequently scheduled for an initial intervention session as part of the program and received a mid-point call approximately three weeks into the intervention to provide support and enhance engagement. All activities and surveys were delivered via automatic email links and were completed online using REDCap. Online surveys were administered at baseline (T0), midpoint (3-weeks, T1), post-intervention (6-weeks, T2), and during two follow-up periods (3-months, T3, and 6-months, T4).

**Statistical analysis**

Analyses were performed using R (version 4.2.1; R Core Team, 2020) and MplusAutomation (Hallquist & Wiley, 2018) on an intention-to-treat basis. No outliers were identified, data was approximately normally distributed.

Latent growth models (LGMs) were estimated using an intercept and two linear slopes (for model fit indices, see Table S3). Slope 1 had loadings constrained to 0, 0.5, and 1 for T^0^ T^1^ T^2^ and 1 for T^3^ T^4^. **Slope 1** tests the main aim of change during the intervention (i.e., baseline to post-intervention). Slope 2 had loadings constrained to 0 for T^0^ T^1^ and 0, 0.5, and 1 for T^2^ T^3^ T^4^. **Slope 2** tests the exploratory aim of change during the follow-up period (i.e., post-intervention to follow-up). The means and variances of the intercept and slopes were freely estimated. The random intercept was allowed to correlate with the slopes. Slope 1 and 2 were not correlated. Residual variances was constrained to equality across time, and residuals were assumed to be uncorrelated, corresponding to an independent, homogenous residual structure. Dummy codes for the three stratification factors (site, cancer stage, and insomnia severity; see protocol Maccora et al., 2022) were created and included as covariates on the intercept and slopes.

Intervention effects were evaluated using dummy codes for CBT-I, BLT, and the CBT-IxBLT interaction, and entered as predictors of the slopes. The interaction was exploratory as the trial was powered to test main effects. No intervention effects were allowed on the intercept at baseline to implement constrained longitudinal data analysis (Coffman et al., 2016). Main effects were calculated for CBT-I (i.e., average effects of all conditions with CBT-I [CBT-I, CBT-I+BLT] versus the average effects of all conditions without CBT-I [BLT, SHE]), as well as BLT (i.e., average effects of all conditions with BLT [CBT-I+BLT, BLT] versus the average effects of all conditions without BLT [CBT-I, SHE]). Missing data were addressed using full-information maximum likelihood estimation (Enders & Bandalos, 2001).

Intervention effects were reported from baseline (T^0^) to mid-point (T^1^) to post-intervention (T^2^) time points (defined hereafter as Slope 1) to evaluate immediate intervention effects, as well as from post-intervention (T^2^) to 3-month (T^3^) and 6-month (T^4^) follow-up time points (defined hereafter as Slope 2) to evaluate changes post-treatment.

Statistical significance was set at α = .05. To control for type 1 error, the two main effect tests (CBT-I, BLT) were adjusted using the false discovery rate (Benjamini & Hochberg, 1995; Verhoeven et al., 2005). Within-group and exploratory interaction results were not adjusted as they are exploratory. A standardized mean difference (SMD) effect size was calculated for differences between-groups and within-groups change based on the standard deviation of the PROPr score at baseline. Exploratory subgroups on cancer stage (metastatic, stage 4) and baseline insomnia severity (clinical insomnia severity, subthreshold insomnia severity) were tested, with reporting focused on main effects across Slopes 1 and 2.

Intervention effects were evaluated using dummy codes for CBT-I, BLT, and the CBT-IxBLT interaction, and entered as predictors of the slopes. The interaction was exploratory as the trial was powered to test main effects. No intervention effects were allowed on the intercept at baseline to implement constrained longitudinal data analysis (Coffman et al., 2016). Main effects were calculated for CBT-I (i.e., average effects of all conditions with CBT-I [CBT-I, CBT-I+BLT] versus the average effects of all conditions without CBT-I [BLT, SHE]), as well as BLT (i.e., average effects of all conditions with BLT [CBT-I+BLT, BLT] versus the average effects of all conditions without BLT [CBT-I, SHE]). Missing data were addressed using full-information maximum likelihood estimation (Enders & Bandalos, 2001).

**Statistical analysis – drivers of change exploratory analyses**

We conducted three exploratory analyses to examine potential drivers of change in HRQoL, as assessed by PROPr scores. These analyses aimed to identify temporal patterns of change, estimate the contribution of sleep and fatigue-related factors, and assess the clinical meaningfulness of observed improvements.

***A. Change Score Calculation***

Change scores were computed to quantify individual-level changes in PROPr across time. For each participant, change scores were derived for the following contrasts:

• Midpoint (T1) minus Baseline (T0)

• Postintervention (T2) minus Baseline (T0)

• 3-month Follow-up (T3) minus Postintervention (T2)

• 6-month Follow-up (T4) minus Postintervention (T2)

These change scores were used in subsequent regression and subgroup analyses to maintain consistent time-dependent intervals throughout the exploratory analyses.

***B. Variance in HRQoL Change Explained by Sleep and Fatigue Variables***

To investigate the extent to which sleep and fatigue-related variables contributed to changes in HRQoL, we conducted a series of linear regressions. At each of the four timepoint contrasts defined above, PROPr change scores were regressed on time-matched change scores in the following predictors:

• Fatigue

• Sleep quality

• Insomnia symptoms (Insomnia Severity Index)

• Sleep-related impairment

The proportion of variance in HRQoL change explained by these predictors was assessed using the Multiple R-squared statistic from each model. This allowed us to examine how strongly changes in sleep and fatigue variables accounted for variation in PROPr scores over time.

***C. Proportion of Participants Achieving Clinically Meaningful Change (MID Analysis)***

To assess the clinical significance of HRQoL improvements, we calculated the proportion of participants who experienced a minimally important difference (MID), defined as a ≥0.04 increase in PROPr scores.

This analysis focused on the immediate intervention period (Baseline [T0] to Postintervention [T2]).

- For the CBT-I Main Effect, participants were grouped based on whether they received CBT-I (CBTI-only and CBTI+BLT) or did not receive CBT-I (e.g., BLT-only or SHE).
- For the BLT Main Effect, participants were grouped based on whether they received BLT (e.g., CBTI+BLT or BLT only) or did not receive BLT (e.g., CBT-I-only or SHE).

We then compared the proportion of participants in each group who met or exceeded the MID threshold.

**Results**

**Model Information of Latent Growth Models**

Table S1. Model Fit Indices

| Chi² (df) | p (Chi²) | CFI | TLI | SRMR | RMSEA | 90% CI RMSEA | p (RMSEA < .05) | AIC | BIC |
| --- | --- | --- | --- | --- | --- | --- | --- | --- | --- |
| HRQoL (PROPr) | | | | | | | | | |
| 49.446 (28) | 0.0075 | 0.955 | 0.928 | 0.051 | 0.061 | [0.031, 0.088] | 0.243 | -869.079 | -779.096 |
| Cancer Stage (Metastatic, Stage 4) Subgroup | | | | | | | | | |
| 29.127 (24) | 0.2154 | 0.970 | 0.956 | 0.076 | 0.065 | [0, 0.137] | 0.362 | -215.888 | -175.320 |
| Clinical Insomnia Severity (ISI ≥ 15) Subgroup | | | | | | | | | |
| 33.311 (24) | 0.0977 | 0.900 | 0.854 | 0.111 | 0.068 | [0, 0.119] | 0.279 | -395.994 | -344.947 |
| Subthreshold Insomnia Severity (ISI ≤ 14) Subgroup | | | | | | | | | |
| 34.420 (24) | 0.0775 | 0.960 | 0.941 | 0.072 | 0.060 | [0, 0.101] | 0.333 | -469.421 | -410.536 |

*Note.* Table values are taken from model output generated using maximum likelihood (ML) estimation. Fit indices include: Chi2 = model chi-square test statistic; df = degrees of freedom; p = p-value for chi-square test; CFI = Comparative Fit Index; TLI = Tucker-Lewis Index; SRMR = Standardized Root Mean Square Residual; RMSEA = Root Mean Square Error of Approximation; 90% CI RMSEA = 90% confidence interval of RMSEA; p < .05 (RMSEA) = probability RMSEA < .05; AIC = Akaike Information Criterion; BIC = Bayesian Information Criterion.

**Participant demographics and clinical characteristics at baseline**

Table S2. Additional Participant Demographic and Clinical Characteristics at Baseline (T0), by Intervention Group

| **Characteristics** | **CBT-I** | **BLT** | **CBT-I+BLT** | **SHE** | **Overall Cohort** |
| --- | --- | --- | --- | --- | --- |
| **1^st^ language spoken as a child*, n (%)*** |  |  |  |  |  |
| English | 40 (72.7%) | 38 (69.1%) | 31 (59.6%) | 44 (77.2%) | 153 (69.9%) |
| Others | 1 (27.3%) | 17 (30.9%) | 21 (40.4%) | 13 (22.8%) | 66 (30.1%) |
| **Number of children, *n (%)*** | 1.34 (1.27) | 1.47 (1.28) | 1.41 (1.22) | 1.42 (1.36) | 1.41 (1.28) |
| **Radiation therapy commenced, *n (%)*** |  |  |  |  |  |
| No | 43 (86.0%) | 46 (86.8%) | 46 (93.9%) | 47 (88.7%) | 182 (88.8%) |
| Yes | 7 (14.0%) | 7 (13.2%) | 3 (6.1%) | 6 (11.3%) | 23 (11.2%) |
| **Hormonal therapy commenced, *n (%)*** |  |  |  |  |  |
| No | 42 (85.7%) | 45 (84.9%) | 36 (70.6%) | 35 (66.0%) | 158 (76.7%) |
| Yes | 7 (14.3%) | 6 (11.3%) | 10 (19.6%) | 13 (24.5%) | 36 (17.5%) |
| Unknown | 0 (0.0%) | 2 (3.8%) | 5 (9.8%) | 5 (9.4%) | 12 (5.8%) |
| **Current biological agent use, *n (%)*** |  |  |  |  |  |
| No | 31 (62.0%) | 40 (75.5%) | 34 (66.7%) | 34 (64.2%) | 139 (67.1%) |
| Yes | 3 (6.0%) | 0 (0.0%) | 1 (2.0%) | 2 (3.8%) | 6 (2.9%) |
| Unknown | 16 (32.0%) | 13 (24.5%) | 16 (31.4%) | 17 (32.1%) | 62 (30.0%) |

*Note.* M = mean; SD = standard deviation; MDN = median, IQR = interquartile range.

**Household income quartiles**

Ranges provided below in bold are the ranges provided in SleepCaRe REDCap surveys. Those provided in brackets “()” are the ranges are based on 2021 Australia Household Income Quartiles (<https://profile.id.com.au/australia/household-income-quartiles>).

Table S3. Income Quartiles used in SleepCaRe

| **Income Quartiles (per year) ^a^** | Ranges provided in SleepCaRe REDCap survieys | Ranges are based on 2021 Australia Household Income Quartile |
| --- | --- | --- |
| 1^st^ | **< $49000** | (~$45085) |
| 2^nd^ | **$49000 - $99000** | (~$45080 - $90480) |
| 3^rd^ | **$99000 - $149999** | (~$90480 - 153000) |
| 4^th^ | **> $150000** | (~$153000) |

**Drivers of Change Exploratory Analyses**

Table S4. Exploratory Analyses: Change Scores, Sleep-Related Variance, and Clinical Meaningfulness

| Panel A: | Change score, M(SD) ^c^ | | | | |
| --- | --- | --- | --- | --- | --- |
| Outcome variable | Midpoint (T^1^)  - Baseline (T^0^) | Postintervention (T^2^) - Baseline (T^0^) | 3moFollowup (T^3^)  - Postintervention (T^2^) | | 6moFollowup (T^4^)  - Postintervention (T^2^) |
| PROPr (+) | 0.04 (0.12) | 0.07 (0.15) | 0.03 (0.16) | | 0.06 (0.14) |
| ISI (-) | -3.78 (4.12) | -5.06 (4.99) | -1.10 (5.03) | | -1.01 (4.59) |
| Fatigue ^a^ (-) | -2.26 (7.88) | -3.90 (9.36) | -2.88 (8.53) | | -3.08 (7.74) |
| Sleep ^a^ (-) | -4.29 (6.80) | -5.43 (7.70) | -0.68 (8.65) | | -0.88 (8.01) |
| SRI ^b^ (-) | -3.44 (6.47) | -4.51 (7.64) | -1.91 (7.84) | | -2.37 (7.13) |
| Social ^a^ (+) | 1.21 (6.55) | 1.74 (6.97) | 2.09 (7.91) | | 3.77 (6.66) |
| Cognitive ^a^ (+) | 1.21 (6.19) | 1.92 (7.15) | 0.29 (6.28) | | 1.88 (7.04) |
| Depression ^a^ (-) | -0.91 (5.79) | -2.31 (6.08) | -0.12 (7.03) | | -0.47 (6.24) |
| Anxiety ^b^ (-) | -0.81 (5.65) | -3.20 (6.53) | -0.84 (7.38) | | -0.93 (6.96) |
| Pain ^a^ (-) | 0.55 (9.62) | -0.96 (10.31) | 0.28 (10.90) | | -0.18 (11.42) |
| Physical ^a^ (+) | 0.10 (5.15) | 0.73 (6.27) | 1.82 (7.58) | | 2.73 (6.49) |
| Panel B: | Proportion (%) of variance explained by sleep and fatigue variables | | | | |
|  | Midpoint (T^1^) - Baseline (T^0^) | Postintervention (T^2^) - Baseline (T^0^) | 3moFollowup (T^3^) - Postintervention (T^2^) | | 6moFollowup (T^4^)  - Postintervention (T^2^) |
|  | 0.5844 | 0.5993 | 0.4848 | | 0.5876 |
| Panel C: | Proportion (%) of sample that had clinically meaningful improvements  (PROPr change ≥ .04) by condition | | | | |
|  | Postintervention (T^2^) - Baseline (T^0^) | | | | |
| CBT-I  Main Effect^d^ | Did not receive CBTI  (i.e., BLT, SHE) | | | Received CBTI  (i.e., CBTI or CBTI+BLT) | |
|  | 44 (50.6%) | | | 52 (71.2%) | |
| BLT  Main Effect^e^ | Did not receive BLT  (i.e., CBTI or SHE) | | | Received BLT  (i.e., BLT or CBTI+BLT) | |
|  | 53 (63.9%) | | | 43 (55.8%) | |

Note. ISI = insomnia severity index, SRI = sleep related impairment. Outcome variable descriptions indicate whether higher (+) or lower (−) scores are favorable.

**^a^** = PROMIS PROPr Subscale

**^b^** = PROMIS CAT

**^c^** = Reported values reflect change scores, rather than raw means. All are T-score changes, except ISI and PROPr, which use raw scale scores and preference-based scores, respectively. **^d^** = Average effects of all conditions with CBT-I (i.e., CBT-I, CBT-I+BLT) versus the average effects of all conditions without CBT-I (BLT, SHE)

**^e^** = Average effects of all conditions with BLT (CBT-I+BLT, BLT) versus the average effects of all

conditions without BLT (CBT-I, SHE)

***Key Takeaways:***

Panel A

- Most gains observed during the intervention period, biggest change in target (sleep/fatigue) domains
- Early changes from baseline to midpoint driven primarily by significant reductions in sleep disturbance (M = -4.29, SD = 6.80), insomnia severity (ISI; M = -3.78, SD = 4.12), fatigue (M = -2.26, SD = 7.88). and sleep-related impairment (M = -3.44, SD 6.47)

Panel B

- Sleep and fatigue-related variables explained a substantial proportion of variance in PROPr change at all timepoints: 58.4% (baseline to midpoint), 59.9% (baseline to postintervention), 48.5% (postintervention to 3-month follow-up), and 58.8% (postintervention to 6-month follow-up).
- This highlights the consistent contribution of sleep and fatigue improvements to overall HRQoL gains. It may also indicate that the remainder of the change is attributable to naturalistic improvements that are not related to sleep, such as changes in cancer treatments, or that sleep-related improvements are having a downstream effect on other non-sleep subdomains of HRQoL.

Panel C

- Panel C shows the proportion of participants achieving clinically meaningful improvement in PROPr (≥ 0.04) from baseline to postintervention by intervention condition.
- A higher percentage of participants who received CBT-I (71.2%) demonstrated meaningful improvement compared to those who did not receive CBT-I (50.6%).
- In contrast, participants receiving BLT showed slightly lower rates of improvement (55.8%) compared to those not receiving BLT (63.9%). These patterns suggest a stronger effect for CBT-I on HRQoL gains than for BLT.

***Additional PROPr Figures***

Figure S1. Observed HRQoL Over Time from Baseline to 6 Month Follow-up

*
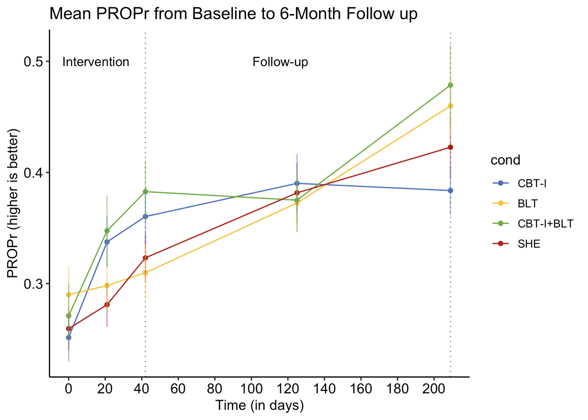
*

Figure S2. Individual Participant Change in HRQoL by Condition

Figure S3. Diagram of Latent Growth Model


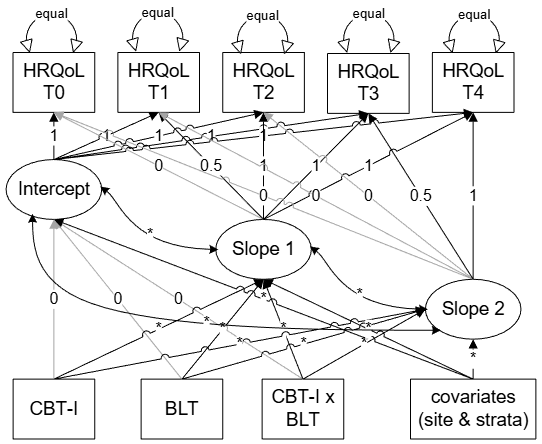


**References**

Benjamini, Y., & Hochberg, Y. (1995). Controlling the False Discovery Rate: A Practical and Powerful Approach to Multiple Testing. *Journal of the Royal Statistical Society: Series B (Methodological)*, *57*(1), 289–300. https://doi.org/10.1111/j.2517-6161.1995.tb02031.x

Clarke, D. E., & Kuhl, E. A. (2014). DSM-5 cross-cutting symptom measures: A step towards the future of psychiatric care? *World Psychiatry*, *13*(3), 314–316. https://doi.org/10.1002/wps.20154

Coffman, C. J., Edelman, D., & Woolson, R. F. (2016). To condition or not condition? Analysing “change” in longitudinal randomised controlled trials. *BMJ Open*, *6*(12), e013096. https://doi.org/10.1136/bmjopen-2016-013096

Dewitt, B., Feeny, D., Fischhoff, B., Cella, D., Hays, R. D., Hess, R., Pilkonis, P. A., Revicki, D. A., Roberts, M. S., Tsevat, J., Yu, L., & Hanmer, J. (2018). Estimation of a Preference-Based Summary Score for the Patient-Reported Outcomes Measurement Information System: The PROMIS®-Preference (PROPr) Scoring System. *Medical Decision Making: An International Journal of the Society for Medical Decision Making*, *38*(6), 683–698. https://doi.org/10.1177/0272989X18776637

Dewitt, B., Jalal, H., & Hanmer, J. (2020). Computing PROPr Utility Scores for PROMIS® Profile Instruments. *Value in Health*, *23*(3), 370–378. https://doi.org/10.1016/j.jval.2019.09.2752

Dickerson, S. S., Connors, L. M., Fayad, A., & Dean, G. E. (2014). Sleep–wake disturbances in cancer patients: Narrative review of literature focusing on improving quality of life outcomes. *Nature and Science of Sleep*, *6*, 85–100. https://doi.org/10.2147/NSS.S34846

Enders, C. K., & Bandalos, D. L. (2001). The Relative Performance of Full Information Maximum Likelihood Estimation for Missing Data in Structural Equation Models. *Structural Equation Modeling: A Multidisciplinary Journal*, *8*(3), 430–457. https://doi.org/10.1207/S15328007SEM0803_5

Gudmundsdottir-Aspelund, S. (2025). *Stress & cognitive function: Exploring the impact of PTSD and breast cancer on cognition and the potential benefit of bright light therapy*. https://opinvisindi.is/handle/20.500.11815/5310

Hallquist, M. N., & Wiley, J. F. (2018). MplusAutomation: An R Package for Facilitating Large-Scale Latent Variable Analyses in Mplus. *Structural Equation Modeling: A Multidisciplinary Journal*, *25*(4), 621–638. https://doi.org/10.1080/10705511.2017.1402334

Hanmer, J., Dewitt, B., Yu, L., Tsevat, J., Roberts, M., Revicki, D., Pilkonis, P. A., Hess, R., Hays, R. D., Fischhoff, B., Feeny, D., Condon, D., & Cella, D. (2018). Cross-sectional validation of the PROMIS-Preference scoring system. *PLOS ONE*, *13*(7), e0201093. https://doi.org/10.1371/journal.pone.0201093

Hung, C.-M., Zeng, B.-Y., Zeng, B.-S., Sun, C.-K., Cheng, Y.-S., Su, K.-P., Wu, Y.-C., Chen, T.-Y., Lin, P.-Y., Liang, C.-S., Hsu, C.-W., Chu, C.-S., Chen, Y.-W., Yeh, P.-Y., Wu, M.-K., Tseng, P.-T., & Matsuoka, Y. J. (2023). Cancer related fatigue—light therapy: Updated meta-analysis of randomised controlled trials. *BMJ Supportive & Palliative Care*, *13*(e2), e437–e445. https://doi.org/10.1136/bmjspcare-2021-003135

Jeste, N., Liu, L., Rissling, M., Trofimenko, V., Natarajan, L., Parker, B. A., & Ancoli-Israel, S. (2013). Prevention of quality-of-life deterioration with light therapy is associated with changes in fatigue in women with breast cancer undergoing chemotherapy. *Quality of Life Research*, *22*(6), 1239–1244. https://doi.org/10.1007/s11136-012-0243-2

Maccora, J., Garland, S. N., Ftanou, M., Day, D., White, M., Lopez, V. A., Mortimer, D., Diggens, J., Phillips, A. J. K., Wallace, R., Alexander, M., Boyle, F., Stafford, L., Francis, P. A., Bei, B., & Wiley, J. F. (2022). The Sleep, Cancer and Rest (SleepCaRe) Trial: Rationale and design of a randomized, controlled trial of cognitive behavioral and bright light therapy for insomnia and fatigue in women with breast cancer receiving chemotherapy. *Contemporary Clinical Trials*, *120*, 106877. https://doi.org/10.1016/j.cct.2022.106877

R Core Team. (2020). *R: A Language and Environment for Statistical Computing* [Computer software]. Vienna, Austria: R Foundation for Statistical Computing. https://www.R-project.org/

Sheehan, D. V., Lecrubier, Y., Sheehan, K. H., Amorim, P., Janavs, J., Weiller, E., Hergueta, T., Baker, R., & Dunbar, G. C. (1998). The Mini-International Neuropsychiatric Interview (M.I.N.I.): The development and validation of a structured diagnostic psychiatric interview for DSM-IV and ICD-10. *The Journal of Clinical Psychiatry*, *59 Suppl 20*, 22-33;quiz 34-57.

Verhoeven, K. J. F., Simonsen, K. L., & McIntyre, L. M. (2005). Implementing false discovery rate control: Increasing your power. *Oikos*, *108*(3), 643–647. https://doi.org/10.1111/j.0030-1299.2005.13727.x

Xiao, P., Ding, S., Duan, Y., Li, L., Zhou, Y., Luo, X., Xie, J., & Cheng, A. S. K. (2021). Effect of Light Therapy on Cancer-related Fatigue: A Systematic Review and Meta-Analysis. *Journal of Pain and Symptom Management*. https://doi.org/10.1016/j.jpainsymman.2021.09.010
